# Corneal Biomechanics After SMILE, Femtosecond-Assisted LASIK, and Photorefractive Keratectomy: A Matched Comparison Study

**DOI:** 10.1167/tvst.12.3.12

**Published:** 2023-03-16

**Authors:** Hassan Hashemi, Cynthia J. Roberts, Ahmed Elsheikh, Shiva Mehravaran, Parsa Panahi, Soheila Asgari

**Affiliations:** 1 Noor Ophthalmology Research Center, Noor Eye Hospital, Tehran, Iran; 2 Department of Ophthalmology & Visual Sciences, Department of Biomedical Engineering, The Ohio State University, Columbus, OH, USA; 3 School of Engineering, University of Liverpool, Liverpool, UK; 4 National Institute for Health Research (NIHR) Biomedical Research Centre for Ophthalmology, Moorfields Eye Hospital NHS Foundation Trust and UCL Institute of Ophthalmology, London, UK; 5 Beijing Advanced Innovation Center for Biomedical Engineering, Beihang University, Beijing, China; 6 School of Computer, Mathematical, and Natural Sciences, Morgan State University, Baltimore, MD, USA; 7 Noor Research Center for Ophthalmic Epidemiology, Noor Eye Hospital, Tehran, Iran

**Keywords:** corneal stiffness, small incision lenticule extraction, femtosecond laser-assisted in situ keratomileusis, photorefractive keratectomy, matched comparison study

## Abstract

**Purpose:**

To evaluate the change in corneal stiffness after small incision lenticule extraction (SMILE), femtosecond laser-assisted in situ keratomileusis (FS-LASIK), and photorefractive keratectomy (PRK).

**Methods:**

Age, gender, spherical equivalent, and central corneal thickness (CCT)–matched cases undergoing SMILE with a 120-µ cap, FS-LASIK with a 110-µ flap, and PRK were enrolled. One-year change in the stress–strain index, stiffness parameter at first applanation, integrated inverse radius, deformation amplitude ratio at 2 mm, and deformation amplitude ratio at 1 mm were compared between the surgical groups by linear mixed-effect models.

**Results:**

Within each surgical group, 120 eyes completed 1 year of follow-up. The residual stromal bed (RSB) thickness and (RSB/CCT_postop_) were 348.1 ± 35.0 (0.74), 375.4 ± 31.0 (0.77) and 426.7 ± 2 µm (0.88) after SMILE, FS-LASIK, and PRK, respectively. The 1-year change in all biomechanical indices was significant, except the stress–strain index with PRK (*P* = 0.884). The change in all indices with SMILE were significantly greater than with FS-LASIK and with PRK (all *P* < 0.01), except the deformation amplitude ratio at 1 mm change between SMILE and FS-LASIK (*P* = 0.075). The changes in all indices with FS-LASIK were significantly greater than with PRK (all *P* < 0.05).

**Conclusions:**

Although SMILE preserves the greatest amount of anterior cornea with a cap thickness of 120 µ, this also produces the smallest RSB and the greatest decrease in stiffness. Thus, the RSB is shown to be the predominant determinant of stiffness decreases, rather than the preserved anterior cornea. We recommend using a thinner cap to achieve a thicker RSB and a lesser decrease in the corneal stiffness in the SMILE procedure.

**Translational Relevance:**

After refractive surgery, RSB is predominant determinant of stiffness decreases, rather than the preserved anterior cornea.

## Introduction

The literature indicates that laser refractive surgery, which is a common corrective approach for refractive errors such as myopia, can change the biomechanical properties of the cornea.^1^^,^^2^ On one hand, baseline corneal biomechanics and stiffness can influence the efficacy and predictability of surgical outcomes, and, on the other hand, surgery-related changes in these parameters can be a precursor of ectasia, which is one of the serious complications of refractive surgery.^3^^–^^5^ Although the characteristics of a patient's cornea and degree of refractive error correction have the greatest impacts on postoperative biomechanical properties, the type of procedure can also be a determinant factor.^2^ Recent studies have suggested that flapless surgeries, including photorefractive keratectomy (PRK), as the traditional surface ablation procedure, and small incision lenticule extraction (SMILE) result in fewer changes in corneal biomechanics than flap-based approaches such as laser-assisted in situ keratomileusis (LASIK).^1^^,^^6^^–^^9^

Corneal Visualization Scheimpflug Technology (Corvis-ST; Oculus Optikgeräte GmbH, Germany) is a noninvasive clinical tool that can assess the stiffness and biomechanical deformation response of the cornea. The Corvis-ST uses an ultra-high-speed Scheimpflug video camera to record corneal movements and deformation in response to a calibrated and focused puff of air.^10^ Dynamic corneal response (DCR) parameters are extracted from the series of images that characterize the magnitude and shape of the corneal deformation. A subset of DCRs has been reported to be related to corneal stiffness^11^ and includes the stiffness parameter at the first applanation event (SP-A1),^12^ the stress–strain index (SSI),^13^ integrated inverse radius (IIR), deformation amplitude ratio at 2 mm (DA ratio-2mm), and deformation amplitude ratio at 1 mm (DA ratio-1mm).^12^ Many DCRs are strongly influenced by the intraocular pressure (IOP), including the magnitude and velocity of deformation.^14^ However, those that characterize the shape of the cornea during deformation have been shown to be related to stiffness.^11^

Numerous studies have investigated changes in Corneal Hysteresis and Corneal Resistance Factor measured by the Ocular Response Analyzer after laser refractive surgery and reported inconclusive results.^1^^,^^6^^,^^15^ However, it is likely that these viscoelastic parameters do not capture the predominant elastic change in stiffness after refractive surgery, because they are affected by both elasticity and viscosity. Furthermore, few studies^16^ have reviewed postoperative changes in the subset of the DCR measured by Corvis-ST or compared only femtosecond-assisted LASIK (FS-LASIK) and PRK.^17^ In addition, to the best of our knowledge, there has been no study on the postoperative 1-year changes in these parameters for any of these three types of surgery.

The aim of this matched comparative study was to assess the biomechanical changes caused by SMILE, FS-LASIK, and PRK using the stiffness-related parameters provided by Corvis-ST. To determine the effect of the residual stromal bed (RSB) thickness on the biomechanical changes, CCT and spherical equivalent were matched between the surgical groups.

## Methods

### Ethical Considerations

The Ethics Committee of Tehran University of Medical Sciences reviewed and approved the protocol of this study (ID: IR.TUMS.MEDICINE.REC.1399.193). After explaining the purpose of the study to the patients, written informed consent for study participation was obtained. The study adhered to the tenets of the Helsinki Declaration at all stages.

### Study Setting and Patients

This prospective matched cohort study was conducted in 2020, and participants were selected from myopic patients undergoing laser refractive surgery. Each candidate underwent a comprehensive preoperative workup including vision tests and ophthalmic examinations to assess eligibility, including those aged 20 years or older and with a stable spherical equivalent (change of less than ±0.5 D) in the past 12 months, and to rule out contraindications such as signs of ectasia and systemic disease. Choice of which surgical procedure to undergo was based on the RSB; the minimum required was 280 µm for SMILE, 300 µm for FS-LASIK, and 350 µm for PRK (with epithelium). All patients were advised not to wear contact lenses for at least 4 weeks before the surgery.

### Participant Enrollment

In the three surgical groups described elsewhere in this article, cases with preoperative myopia −6.0 to −3.0 D and refractive astigmatism of less than 2.0 D who consented to participate were eligible for this study. Patients undergoing SMILE were entered consecutively. For each case of SMILE, one matched case was prospectively enrolled in each of the FS-LASIK and PRK groups. Matching was based on gender, age (±3.0 years), and CCT measured by Pentacam HR (±5.0 µm). Only one eye per participant was enrolled randomly.

### Surgical Techniques

#### Small Incision Lenticule Extraction

All SMILE procedures were done by an ophthalmologist (H.H.) using the VisuMax laser platform (Carl Zeiss Meditec AG, Jena, Germany). After topical anesthesia, patients were instructed to fixate on an internal light source. Lenticule formation parameters were set as incision angle of 52°, incision width of 3.0 mm, and transition zone of 0.1 mm; similar settings were applied. Cap diameter, cap thickness, and optical zone were, respectively, 7.70 mm, 120 µm, and 6.50 mm. For lenticule extraction, the posterior surface of the lenticule was dissected from the periphery to the center, and then its anterior surface was dissected from the center to the periphery. After surgery, patients received chloramphenicol eye drop 0.5% (Sina Darou, Tehran, Iran) four times daily for 3 days, betamethasone eye drops 0.1% (Sina Darou) four times daily for 1 week, and preservative free artificial tears (Hypromellose) four times daily for 1 month.

#### Femtosecond Laser-Assisted In Situ Keratomileusis

After topical anesthesia, Femto LDV (Ziemer Ophthalmic Systems AG, Port, Switzerland) was used for creating a 110 µm thick flap. After lifting the flap, WaveLight Allegretto EX500 (Alcon, TX, USA) excimer laser was used for performing wavefront-optimized ablation. Ablation was done in the 6.50-mm optical zone with a blend zone of 1.25 mm. The postoperative regimen for these patients was chloramphenicol eye drop 0.5% four times daily for 3 days and betamethasone eye drop 0.1% four times daily for 7 days.

#### Photorefractive Keratectomy

After topical anesthesia, the corneal epithelium was scraped mechanically without alcohol. In the second step, the WaveLight Allegretto EX500 excimer laser was used to apply aberration-free ablation to a 6.50-mm optical zone with a 1.25-mm blend zone. After ablation, mitomycin-C 0.02% was applied for 10 seconds per corrected diopter. Then 30 mL of sterile balanced salt solution was used for rinsing the surface of the treated cornea and a bandage contact lens (Ciba Vision, Duluth, GA, USA) was applied. Patients were prescribed betamethasone eye drop 0.1% and levofloxacin eye drop 5 mg/mL four times daily for 1 week, and artificial tears (Hypromellose) as needed. Patients were examined daily until the corneal epithelium was completely healed. After complete epithelial healing, the bandage contact lens was removed and levofloxacin was discontinued, but artificial tears and betamethasone were continued for another 2 weeks. Fluorometholone eye drops 0.1% (Sina Darou) were administered for 3 months with gradual tapering.

### Preoperative and Postoperative Examinations

All patients underwent complete eye examinations, IOP measurement with Goldmann Applanation Tonometry, vision testing, and measurement of corneal biomechanics before surgery and at 3 and 12 months after treatment. The same optometrist used the Snellen SC-2000 system (Nidek Inc., Tokyo, Japan) to determine uncorrected and corrected distance visual acuity and retinoscopy (ParaStop HEINE BETA 200; HEINE Optotechnik, Herrsching, Germany) to determine refraction parameters.

Corneal biomechanical parameters were measured using the Corvis-ST, including biomechanically corrected IOP. All acquisitions were made between 8:00 am and 12:00 noon by the same technician, and those with less than 93.0% data validity were repeated one-half of an hour later. Extracted biomechanical indices included SSI, SP-A1, IIR, DA ratio-2mm, and DA ratio-1mm, which are associated with corneal stiffness and are relatively independent of IOP.^13^^,^^18^

### Statistical Analyses

All analyses were performed using R package version 3.5.2 (R Core Team, Vienna, Austria) and SPSS version 21 (IBM Corp., Armonk, NY, USA). Within each surgical group (SMILE, FS-LASIK, and PRK) changes and between group differences in 1-year changes (∆) were assessed using linear random mixed-effect models. In the analyses, the correlation between matched eyes and the follow-up times were applied in an autoregressive correlation matrix. Generalized estimating equations were used to compare the corneal tissue parameters between surgical groups with accounted for the matched eyes by an unstructured correlation matrix. RSB was calculated by preoperative CCT – (change of CCT + flap/cap thickness) in the FS-LASIK and SMILE groups.

## Results

**Figure 1.**
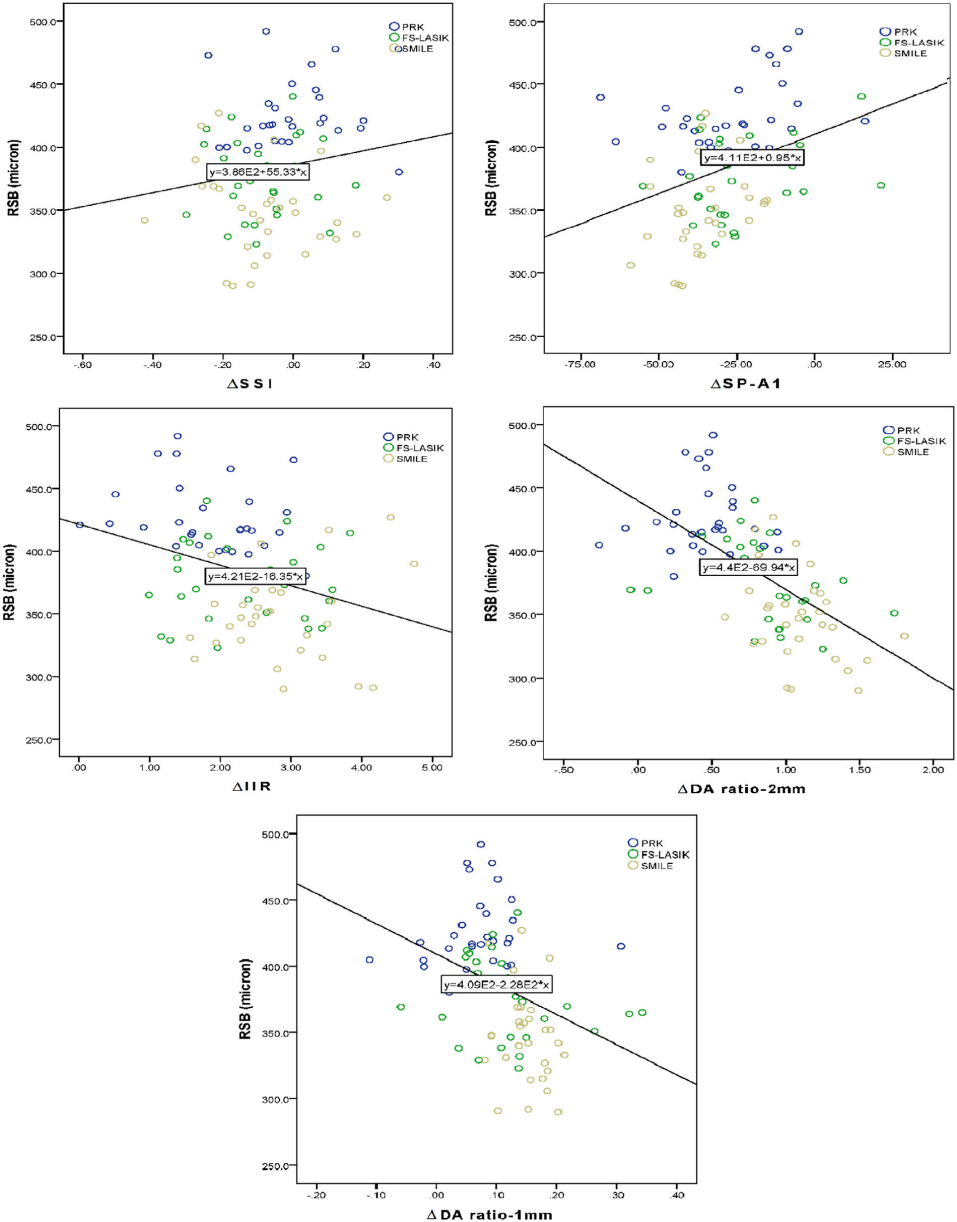
Correlation between RSB thickness and 1-year change (∆) in biomechanical indices after types of refractive surgery. The regression lines are fitted at total cases.

Data from 360 patients with moderate myopia (120 eyes in each surgical group) were used in this analysis. Demographic characteristics, visual acuity, refraction, and biomechanically corrected IOP of this sample are given in Table 1. None of these parameters were significantly different between the three surgical groups (all *P* > 0.05). The corneal tissue parameters including preoperative CCT, postoperative (postop) CCT, ∆CCT, RSB, and RSB/CCT_post__op_ are compared in Table 2. Correlation between RSB thickness and 1-year changes in biomechanical indices after three types of refractive surgery is presented in Figure 1. In this figure, the regression lines are fitted to the total cases.

**Table 1.** Demographic Information and Study Parameters in Moderate (*n* = 360 Eyes) Myopic Patients Treated With SMILE, FS-LASIK, and PRK

	SMILE	FS-LASIK	PRK
No. of eyes	120	120	120
Age (years)	28.03 ± 5.26	29.57 ± 4.89	30.04 ± 6.51
Sex (F)	60.3%	53.6%	51.7%
Preoperative bIOP (mm Hg)	18.17 ± 2.40	17.29 ± 2.91	18.36 ± 3.11
Preoperative MRSE (D)	−4.66 ± 0.85	−4.47 ± 0.82	−4.36 ± 0.72
Postoperative MRSE (D)	0.15 ± 0.34	−0.00 ± 0.33	0.10 ± 0.35
Preoperative UDVA (logMAR)	1.40 ± 0.26	1.37 ± 0.28	1.40 ± 0.14
Post-UDVA (logMAR)	0.01 ± 0.03	0.00 ± 0.00	0.00 ± 0.02
Preoperative CDVA (logMAR)	0.00 ± 0.00	0.00 ± 0.00	0.00 ± 0.00
Postoperative CDVA (logMAR)	0.00 ± 0.01	0.00 ± 0.00	0.00 ± 0.00
Safety index	0.99 ± 0.03	1.00 ± 0.00	1.00 ± 0.00
Efficacy index	0.99 ± 0.02	1.00 ± 0.00	1.00 ± 0.00

bIOP, biomechanically corrected intraocular pressure; CDVA, corrected distance visual acuity; MRSE, manifest refraction spherical equivalent; UDVA, uncorrected distance visual acuity.

**Table 2.** Corneal Tissue Parameters in Moderate Myopic Patients Treated With SMILE, FS-LASIK, and PRK (*n* = 360 Eyes)

	SMILE	FS-LASIK	PRK	SMILE vs. FS-LASIK	SMILE vs. PRK	FS-LASIK vs. PRK
No. of eyes	120	120	120			
Preoperative CCT (µm)	567.03 ± 25.32	564.39 ± 27.52	560.52 ± 25.92	0.864	0.405	0.733
Postoperative CCT (µm)	468.13 ± 34.99	486.75 ± 33.40	485.41 ± 28.85	0.010	0.017	0.976
∆CCT (µm)	−98.90 ± 20.87	−77.64 ± 20.79	−75.10 ± 21.11	<0.001	<0.001	0.811
RSB (µm)	348.13 ± 34.99	375.44 ± 31.02	426.73 ± 27.66	<0.001	<0.001	<0.001
RSB/CCT_postop_	0.74 ± 0.02	0.77 ± 0.02	0.88 ± 0.02	<0.001	<0.001	<0.001

RSB, residual stromal bed; CCT, central corneal thickness; op, operation.

Table 3 demonstrates the biomechanical indices in the SMILE, FS-LASIK, and PRK groups. The baseline biomechanical indices were not significantly different between the three surgical groups (all *P* > 0.05). Within-group analysis showed that the 1-year change in SSI was significant in the SMILE (*P* < 0.001) and FS-LASIK groups (*P* < 0.001), but not in the PRK group (*P* = 0.884). The 1-year change in SP-A1, IIR, DA ratio-2mm, and DA ratio-1mm were significant in all surgical groups (all *P* < 0.001).

**Table 3.** Baseline and Postoperative Stress–Strain and Stiffness Indices in Cases of Moderate Myopia Treated With SMILE, FS-LASIK, and PRK

		Baseline	3 Months	1 Year	*P* Value^*^
SSI	SMILE	1.070 ± 0.170	0.940 ± 0.143	0.954 ± 0.150	<0.001
	FS-LASIK	1.083 ± 0.169	1.034 ± 0.175	1.037 ± 0.175	<0.001
	PRK	1.065 ± 0.134	1.074 ± 0.170	1.062 ± 0.168	0.884
SP-A1	SMILE	117.81 ± 13.91	82.36 ± 16.87	82.54 ± 16.60	<0.001
	FS-LASIK	117.94 ± 13.81	93.07 ± 14.90	93.33 ± 16.64	<0.001
	PRK	119.76 ± 15.34	95.58 ± 15.72	96.16 ± 17.36	<0.001
IIR	SMILE	6.64 ± 0.88	9.65 ± 0.96	9.52 ± 0.95	<0.001
	FS-LASIK	6.63 ± 1.00	9.04 ± 0.78	8.90 ± 0.89	<0.001
	PRK	6.74 ± 0.77	8.82 ± 0.94	8.72 ± 0.99	<0.001
DA ratio-2mm	SMILE	4.05 ± 0.39	5.28 ± 0.55	5.19 ± 0.51	<0.001
	FS-LASIK	4.12 ± 0.36	4.98 ± 0.41	4.97 ± 0.46	<0.001
	PRK	4.09 ± 0.31	4.72 ± 0.40	4.65 ± 0.35	<0.001
DA ratio-1mm	SMILE	1.56 ± 0.06	1.69 ± 0.05	1.69 ± 0.06	<0.001
	FS-LASIK	1.57 ± 0.05	1.67 ± 0.05	1.68 ± 0.07	<0.001
	PRK	1.56 ± 0.05	1.64 ± 0.05	1.63 ± 0.09	<0.001

* One-year changes in the biomechanical indices in each surgical group adjusted for the correlation of follow-up times.

**Figure 2.**
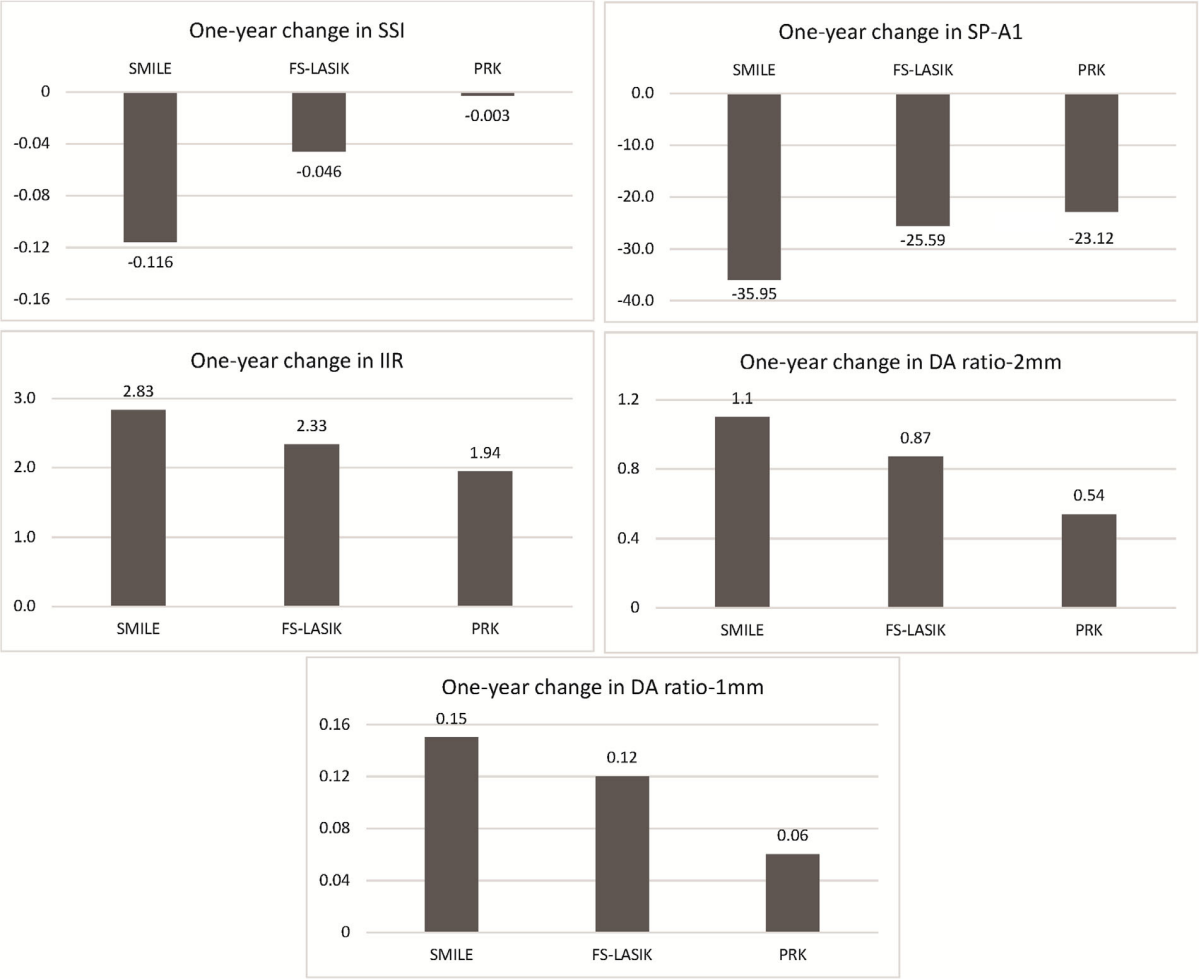
One-year change in the SSI, SP-A1, IIR, DA ratio-2mm, and DA ratio-1mm after SMILE, FS-LASIK, and PRK. One-year changes in the biomechanical indices were significantly different between the three surgical groups, except DA ratio-1mm between SMILE and FS-LASIK (*P* = 0.075).

Between-group analysis demonstrated the ∆SSI, ∆SP-A1, ∆IIR, ∆DA ratio-2mm, and ∆DA ratio-1mm were significantly different between the three surgical groups (all *P* < 0.05), except ∆DA ratio-1mm between SMILE and FS-LASIK (*P* = 0.075) (Fig. 2). The 1-year change in SSI and all DCR parameters with SMILE were significantly greater than with FS-LASIK and with PRK (all *P* < 0.01). The changes in all indices with FS-LASIK were significantly greater than with PRK (all *P* < 0.05).

## Discussion

To date, three systematic reviews of corneal biomechanical changes after refractive surgery, as measured with the Ocular Response Analyzer, have returned inconclusive results.^1^^,^^6^^,^^15^ Guo et al.^1^ suggested that SMILE can be superior to FS-LASIK in this regard, but comparable with PRK. In a review by Damgaard et al.,^6^ the authors concluded that, in terms of biomechanical preservation, as measured with both Ocular Response Analyzer and Corvis-ST, results with SMILE were superior or similar to LASIK. In contrast, in a review by Rævdal et al.,^15^ it was reported that SMILE had a similar effect to LASIK.

In the current study, 1-year changes in DCRs that are known as measures of cornea's overall stiffness^11^ were compared between the three forms of laser refractive surgery. The changes in all biomechanical indices showed decreases in corneal stiffness after the three surgical procedures. However, the change in the SSI after PRK (−0.003 ± 0.137) was not significant. Although the SSI was designed to indicate material stiffness of the cornea with low correlation with the tissue's geometric parameters or the IOP, it seems that the SSI does not capture material stiffness fully when there are two layers of tissue that experience two different biomechanical stress environments (cap/flap vs. stroma). This observation is compatible with the fact that the SSI was developed using corneal numerical models in which no tissue separation (as is experienced in LASIK and SMILE) was simulated.^13^ However, in PRK—the surgery form that is compatible with the assumptions considered in SSI development—where there is one layer of tissue,^19^ the SSI does not show a change in the corneal material stiffness, as expected.

Our results indicated that the decreases in DCRs with SMILE were greater than those observed with FS-LASIK in a matched comparison study. Unlike our results, Xin et al.^20^ showed a greater decrease in stiffness with FS-LASIK than SMILE in low to moderate myopia at 6 months after surgery. This difference can be related to different follow-up times. Also, our result may be due to two contributing factors. First, although all three surgery types had the same optical zone of 6.5 mm, both FS-LASIK and PRK included a 1.25-mm blend zone of ablated tissue, whereas the lenticule removed in SMILE did not include a transition zone. This factor resulted in a wider zone of tissue removal and severed corneal lamellae with a smaller peripheral region of relaxed lamellar segments in both Excimer procedures. The smaller peripheral region leaves less tissue to drive the biomechanical response.^21^ In a contralateral study where SMILE was compared with FLeX, a femtosecond laser procedure in which a flap is created to allow removal of the lenticule, the stiffness in the flap region was reported to be an average of 49.0% lower than the stiffness in the cap region of the fellow eye, with the stress being transferred to the RSB.^22^ Finite element models were created of both preoperative and postoperative corneas so that the stress of increasing IOP could be simulated, highlighting the importance of the RSB in two procedures where the only difference was cap versus flap, with similar lenticules created for tissue removal in both eyes. This process leads to the second potential contributing factor to the greater biomechanical change with SMILE in our cases. The thickness of the cap (in SMILE) was 120 µm and the flap (in FS-LASIK) was 110 µm, with tissue removal from a deeper layer of stroma and less RSB after the SMILE procedure, which was associated with greater corneal weakening in this cohort-matched study. It should be mentioned that the lower RSB target in SMILE group was conventional at the time of study and this, along with the 10-micron difference between the cap and the flap, explains the difference between two procedures. In other words, the role of lower RSB thickness with smaller diameter of tissue removal in SMILE combined to generate the biomechanical changes despite the potentially greater stiffness in the cap versus the flap. This observation also explains why the decrease in the DCRs in PRK was lower than in the other two procedures. Although the anterior stroma, which is stiffer than the posterior stroma,^23^ is removed in PRK, a greater RSB in this surgery has led to superiority of this procedure in terms of stiffness preservation. In line with our results, Xin et al.^20^ showed the smallest overall stiffness decrease at 6 months after transepithelial PRK compared with other procedures.

Several theoretical and clinical studies^24^^,^^25^ have suggested that SMILE preserves the anterior part of the stroma, which provides the highest tensile strength of the cornea, and, therefore, the biomechanical properties of the cornea should be less affected compared with LASIK. Yet, our in vivo 1-year results suggest that, although anterior stromal lamellae are intact during a lenticular resection, the RSB and diameter of tissue removal have a greater influence on postoperative stiffness than the amount of anterior tissue preserved. The inconsistency arises because the mathematical model^23^ did not account for the biomechanical effects of the change in structure with tissue removal, but rather used tensile strength from studies of intact donor globes. In addition, the finite element model^24^ did not account for the reduced stiffness in the SMILE cap that was reported in patient-specific finite element modeling of clinical procedures with known outcomes.^22^ An important clinical study^25^ reported greater biomechanical change with LASIK than SMILE. However, both flap and cap were matched at 90 µm, both of which would have produced a greater RSB than the current study, which had much greater cap and flap thicknesses. This comparison is important because it indicates that, if the cap and flap thicknesses are matched, then LASIK shows greater biomechanical change than SMILE, with presumably similar RSBs. There were also clinical studies that reported no difference between SMILE and LASIK in biomechanical parameters,^26^^,^^27^ but the number of subjects in these studies were fewer than 50 per group with 6 months or less of follow-up, whereas the current study had 120 subjects per group with 1 year of follow-up. Therefore, it is likely that these earlier studies were underpowered to detect a difference. Two additional clinical studies^28^^,^^29^ evaluated the biomechanics of cap thickness in SMILE and were consistent with our results that the thicker flap, which resulted in a thinner RSB and showed greater biomechanical change.

The main strength of the present study is its prospective matched design and larger sample size than previous studies, which makes it possible to compare 1-year changes in biomechanical indices after each type of refractive surgery with sufficient power to detect differences. A limitation of the study was the nonrandom allocation of patients into surgical groups. Given the different eligibility criteria for each surgical method used in this clinic, it was not possible to allocate patients randomly.

Based on the changes in the SSI, SP-A1, and other parameters measured by the Corvis-ST, it can be concluded that the RSB is more important in determining stiffness decreases than the thickness of the cap in SMILE. It was thought that the 120-micron cap was intended to preserve the anterior stroma and the 110-micron flap was intended to minimize the number of lamellae severed in the anterior layer. However, this study shows that the determining factor for the reduction in stiffness is the RSB, not the number of anterior lamellae that are preserved. When a thinner cap was used in a published study,^25^ LASIK showed greater biomechanical weakening than SMILE, which showed that, if RSBs were similar between procedures, SMILE produces a fundamentally stronger result. Further evidence is given by the PRK, the procedure that involved the removal of the anterior layer, but maintained the thickest RSB, in which case there was less of a decrease in biomechanical stiffness of the cornea than in the other two procedures. Based on these findings, we suggest using a thinner cap, and hence a thicker RSB and a lesser decrease in corneal biomechanical strength, in the SMILE procedure.
